# Proactive Control of Emotional Distraction: Evidence From EEG Alpha Suppression

**DOI:** 10.3389/fnhum.2020.00318

**Published:** 2020-08-18

**Authors:** Justin Murphy, Christel Devue, Paul M. Corballis, Gina M. Grimshaw

**Affiliations:** ^1^School of Psychology, Victoria University of Wellington, Wellington, New Zealand; ^2^School of Psychology and Centre for Brain Research, University of Auckland, Auckland, New Zealand

**Keywords:** alpha suppression, attention, emotion, cognitive control, EEG

## Abstract

Biased attention towards emotional stimuli is adaptive, as it facilitates responses to important threats and rewards. An unfortunate consequence is that emotional stimuli can become potent distractors when they are irrelevant to current goals. How can this distraction be overcome despite the bias to attend to emotional stimuli? Recent studies show that distraction by irrelevant flankers is reduced when distractor frequency is high, even if they are emotional. A parsimonious explanation is that the expectation of frequent distractors promotes the use of proactive control, whereby attentional control settings can be altered to minimize distraction before it occurs. It is difficult, however, to infer proactive control on the basis of behavioral data alone. We therefore measured neural indices of proactive control while participants performed a target-detection task in which irrelevant peripheral distractors (either emotional or neutral) could appear either frequently (on 75% of trials) or rarely (on 25% of trials). We measured alpha power during the pre-stimulus period to assess proactive control and during the post-stimulus period to determine the consequences of control for subsequent processing. Pre-stimulus alpha power was tonically suppressed in the high, compared to low, distractor frequency condition, regardless of expected distractor valence, indicating sustained use of proactive control. In contrast, post-stimulus alpha suppression was reduced in the high-frequency condition, suggesting that proactive control reduced the need for post-stimulus adjustments. Our findings indicate that a sustained proactive control strategy accounts for the reduction in both emotional and non-emotional distraction when distractors are expected to appear frequently.

## Introduction

An attentional bias towards emotional stimuli can be advantageous, because they often signal the presence of threats and rewards (Yiend, 2010; Okon-Singer et al., 2013; Pourtois et al., 2013) and so can guide adaptive behavior (LeDoux, 1996). Sometimes, however, we need to ignore emotional stimuli so we can achieve other goals (Lee and Chao, 2012). Effective use of emotional information therefore requires that we strike a delicate balance, being open to interruption when emotional stimuli are potentially important but ignoring them when they are not. Here, we explore the mechanisms of *cognitive control* that allow us to negotiate these competing demands.

We draw on the Dual Mechanisms of Control framework (DMC; Braver et al., 2007; Braver, 2012), which provides an explanation of cognitive control in non-emotional contexts. Within the model, cognitive control operates within either a *reactive* or a *proactive* mode (Braver et al., 2009; Braver, 2012). Reactive control involves recruitment of regulatory processes to correct deviations from goal-directed cognition when conflict is detected. In contrast, proactive control is a more effective but resource-intensive strategy whereby conflict is anticipated and prevented before it can occur. According to the model, the extent to which either strategy is used shifts dynamically, based on motivational and task demands, according to a cognitive cost–benefit analysis (Braver, 2012). Because proactive control requires the active (and costly) maintenance of the current goal, we use reactive control as the default strategy and only shift to proactive control when the benefits outweigh the costs, for example, when conflict is expected or when incentives are available to motivate good performance (Braver et al., 2007; Locke and Braver, 2008; Aron, 2011). Although many applications of the DMC framework address response-level conflicts (e.g., in Stroop or cued response tasks), it can also be applied to the control of perceptual conflicts, for example, between goal-relevant targets and goal-irrelevant distractors (i.e., perceptual distraction; Geng, 2014).

Although the DMC framework accounts for cognitive control in non-emotional contexts, it has rarely been applied to the control of emotional distractors, which might be expected to place extra demands on control mechanisms. Recent work in our lab (Grimshaw et al., 2018; Walsh et al., 2018, 2019) shows that emotional distraction can be well-controlled under conditions that encourage the use of proactive control. One way to promote the use of proactive control is to increase the proportion of trials on which conflicts arise, so that they are expected to occur frequently (Corballis and Gratton, 2003; Neo and Chua, 2006; Geyer et al., 2008; Bugg and Crump, 2012). Using an irrelevant peripheral distractor paradigm (modeled on that described by Forster and Lavie, 2008), we showed that increased distractor frequency yields less distraction, even when distractors are emotional (Grimshaw et al., 2018). Participants completed a simple (i.e., low load) letter discrimination task near fixation, while a peripheral distractor image was presented on either 25% or 75% of trials. In separate blocks, distractors could be either high arousal negative images (mutilations), high arousal positive images (erotic couples), or neutral images of people engaged in everyday activities. When distractors appeared on 25% of trials, emotional images slowed response times significantly more than neutral images; however, when distractors appeared on 75% of trials, neither emotional nor neutral images produced any distraction at all (see also Micucci et al., 2020).

A parsimonious explanation of these findings is that high distractor frequency encouraged a shift to proactive control, which effectively reduced emotional distraction. Although behavioral findings are consistent with this explanation, it is also possible that increased distractor frequency facilitated or speeded reactive control processes like distractor suppression, disengagement, or reorienting to the target (Geng et al., 2019). More direct markers of the time course and nature of control can be provided by neuroimaging and psychophysiological evidence (e.g., Chatham et al., 2009; Burgess and Braver, 2010; Jimura et al., 2010; Chiew and Braver, 2013; Botvinick and Braver, 2015; Chevalier et al., 2015; Walsh et al., 2019). For example, behavioral markers of reactive control have been associated with post-conflict increases in pupil dilation (an index of cognitive effort; Chatham et al., 2009; Walsh et al., 2019), and with transient post-stimulus activation of the lateral prefrontal cortex (PFC) and anterior cingulate cortex (ACC)—areas thought to subserve conflict monitoring and resolution processes (Braver et al., 2003). In contrast, behavioral markers of proactive control (e.g., reduced interference following predictive or incentive cues) have been associated with an increase in tonic pupil dilation—indicating sustained use of proactive control across trials—and with phasic dilation in the pre-stimulus period, indicating the dynamic use of proactive control (Chiew and Braver, 2013; Walsh et al., 2019). Neuroimaging studies also show sustained activity in lateral PFC under conditions that encourage proactive control (Burgess and Braver, 2010; Jimura et al., 2010; Marini et al., 2016), reflecting the ongoing active maintenance of task goals.

Posterior-occipital alpha (8–13 Hz) is an alternative online index of cognitive control that has greater temporal sensitivity than either fMRI or pupillometry and therefore may be even better suited for tracking dynamic changes in control in real time. Posterior alpha is established as an inverse measure of cortical excitability (Laufs et al., 2006; Haegens et al., 2011) and attentional engagement (Macdonald et al., 2011; Boudewyn and Carter, 2017; Itthipuripat et al., 2017). Importantly, alpha reflects not just spontaneous fluctuations in attention, but also top-down adjustments of cognitive control (Thut et al., 2006; Capotosto et al., 2009; Carp and Compton, 2009). For example, alpha is suppressed in the pre-stimulus interval following task switching cues, indicating that it is involved in proactive rule updating (Cooper et al., 2016). Alpha suppression is also typically observed post-stimulus and is pronounced following errors or high-conflict stimuli (Compton et al., 2011; Jiang et al., 2015, 2018a,b; Itthipuripat et al., 2017), suggesting that it reflects post-stimulus task engagement as well.

Furthermore, the scalp distribution of alpha modulation is linked to the direction of spatial attention (Frey et al., 2015; Foster and Awh, 2019). In cued attention tasks, alpha shows greater suppression contralateral to the cued visual field (e.g., Sauseng et al., 2005; Kelly et al., 2006; Thut et al., 2006; van Diepen et al., 2016; but see Limbach and Corballis, 2017 for qualification), and this effect is further enhanced by increasing the proportion of valid cues (Gould et al., 2011; van Ede et al., 2012; Dombrowe and Hilgetag, 2014). This lateralization of pre-stimulus alpha has been suggested to reflect top-down anticipatory biasing of cortical excitability in order to enhance potential target locations while inhibiting potential distractor locations (Foxe and Snyder, 2011). The retinotopic modulation of pre-stimulus alpha power is therefore one neural mechanism by which proactive control might be implemented.

Following our previous behavioral work (Grimshaw et al., 2018), we hypothesize that proactive control will be engaged when people anticipate more frequent distractors. We therefore measured EEG while participants completed an irrelevant-distractor task in which they identified a target presented on the vertical midline while ignoring emotional and neutral images presented laterally. Participants were randomly assigned to see distractors on either 25% or 75% of trials. We were interested in two types of proactive control: the sustained maintenance of control across trials, as well as a dynamic enhancement of attentional engagement following a warning signal (a fixation cross) for an upcoming trial. We therefore measured the tonic level of unbaselined alpha observed across a block of trials (as a measure of sustained proactive control), the phasic drop in alpha during preparation for an upcoming trial (measured relative to a pre-fixation baseline; a measure of dynamic proactive control), and an index of alpha lateralization (a measure of location specific control). Additionally, we measured alpha in the post-stimulus period to examine the downstream consequences of proactive control on subsequent processing.

Using these measures, we address four questions: first, does high distractor frequency in the irrelevant-distractor paradigm promote the use of proactive control? If so, then pre-stimulus posterior alpha should be suppressed in the high relative to low distractor frequency condition (either phasically, tonically, or both) reflecting greater task-engagement when preparing for distractors that are expected to appear frequently.

Second, is such control tailored to the expected valence of a distractor? Given that emotional distractors are particularly potent, do participants up-regulate control when emotional distractors are expected? To answer this question, we blocked trials by distractor valence, so that participants could anticipate the type of distractor they might encounter. If people use greater cognitive effort to ignore anticipated emotional (compared to neutral) distractors, then greater pre-stimulus alpha suppression would be expected in emotional relative to non-emotional blocks. We included both positive and negative emotional distractors in order to determine whether the use of control differs according to valence.

Third, is proactive control achieved through the mechanism of location-based distractor inhibition? A modification to the original paradigm used in Grimshaw et al. (2018) allows us to examine pre-stimulus alpha lateralization, which has been proposed to reflect inhibition within the ipsilateral field (Foxe and Snyder, 2011; Vissers et al., 2016; Wildegger et al., 2017). We blocked distractor visual field (left/right) so that participants could anticipate the visual field of an upcoming distractor, although not its specific location within that field. If proactive control is implemented through location-based distractor inhibition (Gaspelin and Luck, 2018), then pre-stimulus alpha should be potentiated contralateral to the expected distractor visual field in the high compared to the low distractor frequency condition.

Finally, we ask how high distractor frequency affects the subsequent neural response to distractors themselves. Greater use of proactive control (prior to stimulus onset) predicts less need for reactive control (following stimulus onset). We addressed this question by examining the effect of distractor frequency and valence on post-stimulus alpha suppression.

## Materials and Methods

### Participants

The effect of distractor frequency on behavioral distraction (measured in RT) in Grimshaw et al. (2018) was *d* = 0.78 (averaged across two experiments), which indicates 27 participants per condition to detect a similar effect with 80% power. For counterbalancing purposes, we ran 60 participants (27 men; 33 women) ranging from 18 to 27 years of age (*M* = 21.78 years, *SD* = 2.87), recruited from an undergraduate psychology pool. One participant was excluded due to discomfort, which prevented them from completing the experiment. All participants were fluent English speakers with normal or corrected-to-normal vision and reported that they were not receiving current treatment for depression or anxiety. Participants were randomly assigned to low or high distractor frequency conditions. They received course credit or movie vouchers in exchange for their participation and provided written informed consent prior to participation. The study was approved by the Human Ethics Committee of the School of Psychology, Victoria University of Wellington (New Zealand).

### Materials

#### Task Procedure

**Figure 1:**
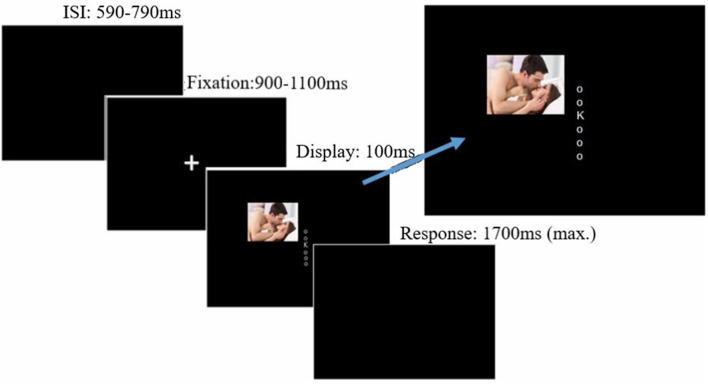
Trial procedure and stimulus display. Distractors could appear lateral to the target display either in the upper or lower quadrant, blocked by hemi-field. Image for illustrative purposes only; the images were drawn from the International Affective Picture System (IAPS; Lang et al., 2008). Displays not to scale. Source: available from https://www.123rf.com/photo_29940472stock-photo.html, © 123rf.com.

The irrelevant-distractor task was very similar to that described in Grimshaw et al. (2018), with some modifications that allowed us to examine lateralized alpha power (see Figure 1). Participants discriminated whether a briefly (100 ms) presented capitalized target letter was a “K” or an “N.” The target letter (font: Arial; font size: 24; color: white; subtending 0.67° of visual angle) was randomly presented in one of six possible locations arranged in a central column along the vertical midline. Letters were evenly spaced (0.67° visual degrees apart), and the top and bottom letters appeared 1.68° of visual angle directly above and below fixation. Lowercase “o”s (font: Arial; font size: 8; subtending 0.22° of visual angle) appeared in the five non-target positions on each trial. Each trial began with a central fixation cross of a random duration between 900 and 1,100 ms. Following fixation, the visual letter display was presented for 100 ms. Participants indicated whether the target was an “N” or a “K” by pressing “1” or “2” with the index and middle finger of their dominant hand. Key response mappings were counterbalanced across participants. On a specified proportion of trials (25% in the low and 75% in the high distractor frequency conditions), a lateralized distractor image was presented simultaneously with the visual letter display, randomly in either an upper or lower quadrant, with the center of the image appearing 7.59° visual angle from fixation.

Participants received auditory feedback (a 100-ms “beep”) following either an incorrect response or a response slower than the 1,700-ms response window. A jittered inter-trial interval was used, ranging between 590 and 790 ms after each response until the onset of the next fixation cross. Trials were blocked by distractor valence (positive, negative, and neutral). Specifically, participants completed three superblocks (192 trials each), in which the distractor was always the same valence, with order counterbalanced across participants. Each valence superblock comprised four sub-blocks of 48 trials each; across sub-blocks, the distractor visual field alternated (left/right or right/left; counterbalanced across participants) every 24 trials. The order of sub-blocks was consistent across each valence-block (i.e., it was always either left/right or right/left throughout the task). Thus, the side of the potential distractor (although not the quadrant) was predictable on each trial. Participants were not told the frequency with which distractors would be presented or that the valence of distractors would alter between blocks; however, they completed two initial practice blocks of 48 trials (four sub-blocks of 12 trials each), with distractor stimuli consisting of pixel scrambles of the intact images used during the task, presented at the same frequency as the assigned condition. This allowed participants to develop an expectancy about distractor frequency and the consistency of the distractor visual field before exposure to intact images.

#### Picture Stimuli

The same distractor stimuli were used as in Grimshaw et al. (2018; see Supplementary Material 1.1 for specific images). Two gender-tailored sets of 36 color images were taken from the International Affective Picture System (IAPS; Lang et al., 2008), selected to have similar mean valence and arousal ratings for each gender. Stimuli were 12 neutral pictures (scenes depicting people in daily life activities), 12 negative pictures (body mutilations), and 12 positive pictures (erotic images involving heterosexual couples). Pictures were matched for luminance and contrast with MATLAB SHINE toolbox (Willenbockel et al., 2010). Pictures subtended 6.68° × 6.68° of visual angle and were presented in color.

### Procedure

Each session took approximately 100 min, including EEG setup. After setup, participants completed the experiment in a dimly lit, electrically shielded room with the task presented on a Dell Precision T1600 computer, with a 23” Alienware 2310 LCD monitor running at a vertical refresh rate of 120 Hz. A viewing distance of 57 cm was maintained using a chinrest. Stimulus presentation and response collection were controlled using E-prime 2.0 software (Psychology Software Tools Inc, 2012). In accordance with our standard lab procedure, before participants received task instructions, they were instructed to rest (with eyes open and with minimum movement) for 2 min, while we recorded their resting EEG activity. Alpha activity during this pre-task resting period did not differ between groups, *t*_(57)_ = 0.086, *p* = 0.932 (see Supplementary Material 1.2).

### EEG Recording

EEG was recorded with a Lycra Quick-Cap (Compumedics NeuroMedical Supplies) embedded with Ag/AgCl electrodes at 28 scalp sites (FP1, FP2, F7, F3, FZ, F4, F8, FT7, FC3, FC4, FT8, T7, C3, CZ, C4, T8, TP7, CP3, CP4, TP8, P7, P3, PZ, P4, P8, O1, Oz, and O2, according to the modified 10-20 system; American Electroencephalographic Society, 1994). To detect blinks and eye movements, the electrooculogram (EOG) was recorded from electrodes placed at the outer canthus of each eye (horizontal), and above and below the left eye (vertical). Electrodes were also placed on the mastoid bones behind the right and left ears. The EEG and EOG channels were referenced online to the left mastoid and re-referenced offline to the algebraic average of the left and right mastoids. Impedances were periodically checked between blocks and were kept below 5 kΩ.

The EEG signal was amplified with Professional BrainAmps and digitized at a sampling rate of 500 Hz with Brain-Vision Recorder (Brain Products, Gilching, Germany). Data were filtered online with a high-pass filter of 0.02 Hz. Signals were analyzed using Brain-vision Analyzer 2.0 (Brain Products, Gilching, Germany). Horizontal EOG (HEOG) and Vertical EOG (VEOG) channels were created by calculating the difference between HEOG-Right and HEOG-Left, and VEOG-Lower and VEOG-Upper, respectively. Data were filtered offline with a low cutoff of 0.01 Hz and a high cutoff of 30 Hz, with a notch filter at 50 Hz using a zero phaseshift Butterworth filter (12 dB/oct). Raw data were manually inspected, and noisy segments were excluded prior to ICA calculation. Ocular artifacts were removed using the ICA ocular correction method in Brainvision Analyzer. The topography of the rejected ICA ocular components was each verified by visual inspection.

### EEG Analysis

For the analysis of pre-stimulus measures, data were partitioned into 2,700-ms epochs, beginning 600 ms before the onset of the fixation cross (i.e., during the ITI) and finishing 2,100 ms after fixation onset (i.e., 1,000–1,200 ms after target display onset). For the analysis of post-stimulus measures, data were segmented into 2,100-ms epochs, beginning 600 ms before stimulus onset (i.e., during the pre-stimulus interval) and finishing 1,500 ms after stimulus onset. These long epochs provide sufficient buffer zones at the beginning and end of each segment to avoid contaminating data in the time windows of interest due to “edge artifacts” produced by the wavelet analysis (Cohen, 2014).

Because we were interested in assessing whether distractor frequency affected alpha power across trials (i.e., tonic alpha), we did not use a baseline correction procedure. Only correct trials were used for analysis. To remove artifacts due to muscle movements, segments with a change in voltage exceeding ±100 μV over posterior electrodes (01, 02, 0z, Pz, P3, P4, P7, and P8) were excluded. This rejection criterion led to a mean total rejection of 4.9% (*SD* = 4.7) of trials. We did not exclude trials in which blinks occurred, as this would have resulted in excessive data loss given the long windows. However, any distortion in posterior alpha due to EOG activity is likely to be negligible (Hagemann and Naumann, 2001) and not systematically associated with condition.

### Alpha Power Analysis

Single-trial power (μV^2^) was estimated separately for the pre-stimulus and post-stimulus epochs, using a Continuous Morlet wavelet transformation, and averaged separately for each valenced superblock. The Morlet time–frequency analysis consisted of 20 linearly spaced frequency bands, ranging from 1 to 20 Hz (Cohen, 2014). To account for individual differences in peak alpha frequency, the specific band used for each individual (e.g., Klimesch, 1999; Pfurtscheller and Da Silva, 1999; Başar, 2012) was chosen based on their individual alpha frequency (IAF), identified in a Fast Fourier Transform across all pre-stimulus epochs, with a frequency resolution of 0.49 Hz, measured at electrodes 01, 0z, and 02 (see Gould et al., 2011 for a similar approach). The frequency band with the closest central frequency to each participant’s IAF was selected for statistical analysis. The mean IAF was 10.06 Hz (SE = 0.12), similar to alpha bands used in other studies (e.g., Limbach and Corballis, 2017). Each participant’s alpha power was pooled across 01, 0z, and 02, averaged separately for each valence condition and at each time point, and exported to R for statistical analysis. Because we were interested in tonic alpha suppression (i.e., across trials), we did not use a baseline correction. To correct for non-normality, mean alpha power values were log-transformed prior to being further analyzed. Shapiro-Wilks tests confirmed that, after transformation, there were no violations of normality in either the pre-stimulus or post-stimulus epochs (all *p*’s > 0.10).

We also calculated a lateralized measure of alpha power for each valence block, comparing sub-blocks in which participants expected distractors to appear in the left and right visual fields. On the pre-stimulus epochs, alpha lateralization indices (ALIs) for the P7/P8 electrode pair were calculated using the formula *(Ipsilateral − Contralateral)/(Ipsilateral + Contralateral)* (Thut et al., 2006). Values of this ALI can vary from −1 to +1, where a negative value indicates greater alpha power over the side contralateral to the potential distractor location (indicating suppression at potential distraction locations), and a value of zero signifies the absence of lateralized differences.

### Statistical Analyses

#### Behavioral Data

Accuracy and mean reaction time (RT) were calculated for each condition. For RT measures, only correct responses with an RT longer than 200 ms were analyzed, ensuring anticipatory responses were not included in the analyses. This exclusion criterion led to the average removal of 3.15% (*SD* = 4.50) of trials per participant. Because our primary hypotheses concerned distraction in terms of increased response latency, a distraction index was calculated for each valenced block as (*RT distractor present − RT distractor absent*). To assess the effects of distractor frequency, distraction indices and accuracy were analyzed in a 3 (valence: negative, neutral, positive) × 2 (distractor frequency: low, high) mixed ANOVA. In order to determine whether distraction was affected by the type of distractor that was expected, we assessed effects of valence (which indicate differences across the three valence conditions) as well as quadratic effects of valence (which indicate differences between emotional and neutral conditions)^1^. Hypothesized interactions between valence and distractor frequency were followed up with one-way ANOVAs in each frequency condition if *p* < 0.10.

#### Pre-stimulus Alpha Power

We first used a mass univariate approach to identify the time windows within the pre-stimulus period in which alpha power was influenced by the expected frequency or valence of upcoming distractors. Non-baselined alpha power was analyzed in a 2 (distractor frequency: low, high) × 3 (valence: negative, neutral, positive) mixed ANOVA at each sampled time point between −200 ms and 1,200 ms (relative to fixation onset). The false discovery rate (FDR) control procedure was used in order to correct for multiple comparisons (Benjamini and Hochberg, 1995; Lage-Castellanos et al., 2010; Groppe et al., 2011; Luck, 2014). Because this mass univariate analysis cannot explicitly assess changes in alpha power across time, we conducted an additional analysis in order to compare alpha power at the beginning and end of the fixation period. Average alpha power was extracted from two time windows: a 200-ms pre-fixation window (i.e., during the ITI) and a 200-ms pre-stimulus window between 700 and 900 ms following the onset of fixation (i.e., immediately prior to the earliest possible onset of a target display). The extracted alpha power was analyzed in a 2 (distractor frequency: low, high) × 2 (time window: pre-fixation, pre-stimulus) × 3 (valence: negative, neutral, positive) mixed ANOVA. We expected to see a drop in alpha power over time (i.e., alpha suppression), consistent with preparation for the upcoming trial. In this analysis, a main effect of distractor frequency would reflect tonic alpha suppression (i.e., sustained proactive control), while an interaction of distractor frequency and time would reflect phasic alpha suppression (i.e., dynamic proactive control). Interactions with valence in either analysis would indicate tailoring of control to the expected valence of an upcoming distractor. As with the behavioral data, we tested both overall and quadratic effects of valence.

#### Alpha Lateralization

To determine whether alpha power was lateralized in anticipation of distractor onset, each ALI (averaged across the pre-stimulus time window) was tested against zero in a Bonferroni-corrected one sample *t*-test. Effects of distractor frequency and valence on alpha lateralization were then analyzed with mass univariate 3 (valence: negative, neutral, positive) × 2 (distractor frequency: low, high) mixed ANOVAs. A main effect of distractor frequency (that is, greater lateralization with high than with low distractor frequency) would indicate proactive inhibition of anticipated distractor location; interactions between distractor frequency and valence would indicate tailoring of inhibition to the expected valence of a distractor.

#### Post-stimulus Alpha Power

To determine whether distractor frequency alters the neural response to distractors, we conducted mass univariate 2 (distractor frequency: low, high) × 2 (distractor present: present, absent) × 3 (valence: negative, neutral, positive) ANOVAs (FDR-corrected) for each data point from stimulus onset until 1,700 ms post-stimulus. As in our analysis of behavioral distraction, we calculated an index of distractor-driven suppression (*Distractor* *Present Alpha − Distractor Absent Alpha)* for further analysis with mass univariate 3 (valence: negative, neutral, positive) × 2 (frequency: high, low) ANOVAs for each data point.

In all analyses, Greenhouse–Geisser corrections were applied when Mauchly’s test of sphericity was violated. Effect sizes are $\eta_{\text{p}}^{2}$ for ANOVA effects, *d*_s_ for comparison of independent means, and *d*_z_ for comparison of paired mean (Lakens, 2013).

## Results

### Behavioral Results

**Figure 2:**
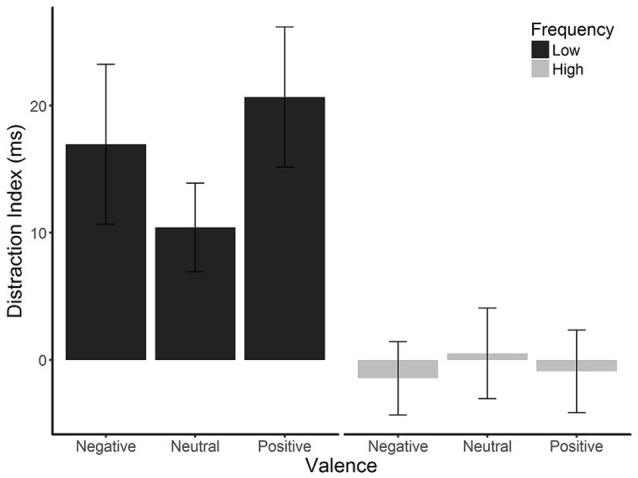
Mean distraction indices (RT on distractor-present trials − RT on distractor-absent trials) by distractor frequency and valence. Emotional distraction was observed in the low distractor frequency condition but not the high distractor frequency condition. Error bars are SEMs corrected for within-subjects comparisons (Morey, 2008).

Mean overall accuracy rates, RTs, and distraction indices are presented in Table 1. Mean RTs were first analyzed in a 2 (distractor frequency: high, low) × 2 (distractor presence: present, absent) × 3 (valence: positive, neutral, negative) ANOVA, which showed no main effect of distractor frequency, *F*_(1,57)_ = 0.056, *p* = 0.813, $\eta_{\text{p}}^{2}$ = <0.01, indicating that there was no overall difference in RTs between conditions. We therefore focused on distraction indices (*RT present − RT absent*) in subsequent analyses, as these are most relevant to hypotheses (see Supplementary Material 1.3 for analyses using RT as the dependent variable). The two-way mixed ANOVA of distractor frequency × valence (see Figure 2) showed the predicted main effect of distractor frequency, *F*_(1,57)_ = 15.65, *p* < 0.001, $\eta_{\text{p}}^{2}$ = 0.215; distraction was attenuated in the high compared to the low distractor frequency condition, consistent with the predictions of the DMC framework. There was no main effect of valence, *F*_(1.68,95.95)_ = 0.663, *p* = 0.493, $\eta_{\text{p}}^{2}$ = 0.011. However, the predicted interaction between distractor frequency and the quadratic effect of valence approached significance, *F*_(1,57)_ = 3.909, *p* = 0.052, $\eta_{\text{p}}^{2}$ = 0.064^2^. Replicating previous findings (Grimshaw et al., 2018), follow-up one-way ANOVAs revealed a significant quadratic effect of valence in the low distractor frequency condition, *F*_(1,28)_ = 5.03, *p* = 0.033, $\eta_{\text{p}}^{2}$ = 0.152, with both positive and negative images producing more distraction than neutral ones. However, there was no effect of valence, nor was there a quadratic effect of valence in the high distractor frequency condition, *F*_(2,58)_ = 0.13, *p* = 0.880, $\eta_{\text{p}}^{2}$ = 0.004, and *F*_(1,29)_ = 0.24, *p* = 0.631, $\eta_{\text{p}}^{2}$ = 0.01, respectively. As expected, accuracy rates were very high overall (*M* = 96.8%, *SD* = 3.9%), and their analysis produced no significant main effects or interactions.

**Table 1:** Mean (SD) response times and distractor indices (in ms) in each experimental condition.

Condition	Distractor present	Distractor absent	Distraction index	*t*	*d*_z_	*p*
**Low frequency**					
Positive	529 (71)	509 (58)	20 (30)	3.7**	0.70	<0.001
Neutral	526 (68)	516 (63)	10 (19)	3.0*	0.53	0.003
Negative	539 (89)	522 (77)	17 (34)	2.7*	0.50	0.006
**High frequency**			
Positive	525 (82)	526 (78)	−1 (18)	0.28	0.06	0.391
Neutral	532 (80)	531 (78)	1 (19)	3.0	0.05	0.442
Negative	527 (81)	529 (81)	−2 (16)	0.50	0.13	0.309

Note: *t*, *d*_z_, and *p*-values refer to the comparison of distractor present and distractor absent trials in each condition. *d*_z_ are effect sizes for within-subject comparisons (Lakens, 2013). *p < 0.05, **p < 0.001.

### EEG Alpha Power

**Figure 3:**
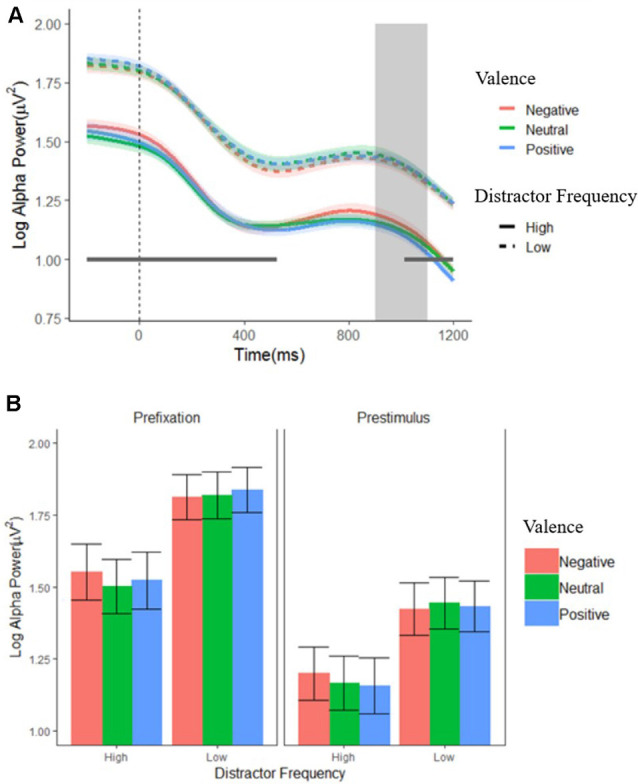
Log alpha power. **(A)** Grand average waveforms of log-transformed individual alpha frequency (IAF) alpha power from pooled electrodes 01, 02, and 0Z in the low (dashed lines) and high (solid lines) distractor frequency conditions, according to valence block. Fixation onset occurs at 0 ms. The gray box indicates the period when a stimulus display could occur (900–1,100 ms). The gray horizontal line indicates where alpha power was significantly reduced in the high compared to low distractor frequency condition. Shaded error bars depict the ±95% within-subjects confidence intervals. **(B)** Average alpha power during the pre-fixation (−200 to 0 ms) and pre-stimulus (700–900 ms) time windows. Error bars depict the between-subjects SEM.

Figure 3 shows alpha power over the entire pre-stimulus time period, for each condition. Time periods during which each effect was significant for each ANOVA are marked on the figure; a complete set of figures showing the *F* and FDR-corrected *p*-values for all effects across the time window appears in Supplementary Figure S1. There was an enduring main effect of distractor frequency, showing that alpha power was tonically suppressed in the high compared to low distractor frequency condition, consistent with the engagement of proactive control. This effect was significant at each data point (*F*’s = 4.75–8.48, *p*’s = 0.039–0.049), except during the 526- to 1,000-ms window, where each data point fell just above conventional levels of significance (*F*’s = 3.905–4.75, *p*’s ≤ 0.053). There were no significant effects or interactions involving valence for any time period.

Consistent with the mass univariate ANOVA results, the ANOVA using just the two time windows (pre-fixation and pre-stimulus) on alpha power also revealed a main effect of distractor frequency, *F*_(1,57)_ = 5.22, *p* < 0.026, $\eta_{\text{p}}^{2}$ = 0.084, showing that alpha was tonically suppressed in the high compared to the low distractor frequency condition. In addition, there was a predicted main effect of window*, F*_(1,57)_ = 102.23, *p* < 0.001, $\eta_{\text{p}}^{2}$ = 0.642, showing that alpha power dropped following fixation cross onset, reflecting preparation to attend to the target. However, there was no time window × distractor frequency interaction, *F*_(1,57)_ = 0.263, *p* = 0.610, $\eta_{\text{p}}^{2}$ = 0.005, suggesting that the phasic changes in alpha (i.e., suppression in response to fixation onset) did not differ between the high and low distractor frequency conditions. Although a significant valence × frequency interaction, *F*_(2,110)_ = 4.45, *p* = 0.014, $\eta_{\text{p}}^{2}$ = 0.072, was present, *post hoc* paired *t*-tests found no significant differences between distractor valences in either condition, *t*’s = −2.47–2.79, *p’*s > 0.066–0.780, suggesting that any differences in alpha suppression according to valence are minimal. Visual inspection of Figure 3 supports this conclusion.

Taken together, our findings indicate that alpha in the high-frequency condition was tonically suppressed across trials (i.e., even before fixation cross onset), but that the phasic drop in alpha following fixation (and in preparation for the upcoming trial) was not sensitive to distractor frequency. In both frequency conditions, there was no notable adjustment of either tonic or phasic alpha suppression according to the expected valence of a distractor.

**Figure 4:**
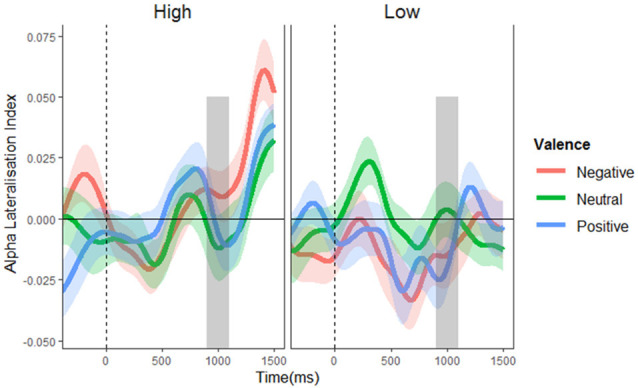
Alpha lateralization indices. Alpha lateralization indices for the low and high distractor frequency conditions, as a function of valence block. A negative value indicates greater alpha power over the side contralateral to the potential distractor location (indicating suppression at potential distraction locations), with a value of zero signifying the absence of lateralized differences. The gray box indicates the time window of possible stimulus onset. No significant alpha lateralization was found.

To test for alpha lateralization, one-sample *t*-tests were conducted on the mean ALI in the pre-stimulus (700–900 ms) period for each valence per condition, using a Bonferroni-corrected alpha level of 0.008. This analysis found no evidence of lateralization in any condition (*t*’s = −1.927–1.509, *p’*s = 0.060–0.613). This finding suggests that proactive suppression of potential distractor locations cannot account for the effective control of distraction observed in the high distractor frequency condition (at least not as implemented by alpha lateralization). Not surprisingly, the mass univariate analysis of ALIs revealed no significant main effects or interactions (*F*’s ≤ 1.857, *p*’s ≥ 0.130; see Figure 4), showing that alpha lateralization did not systematically differ between conditions.

**Figure 5:**
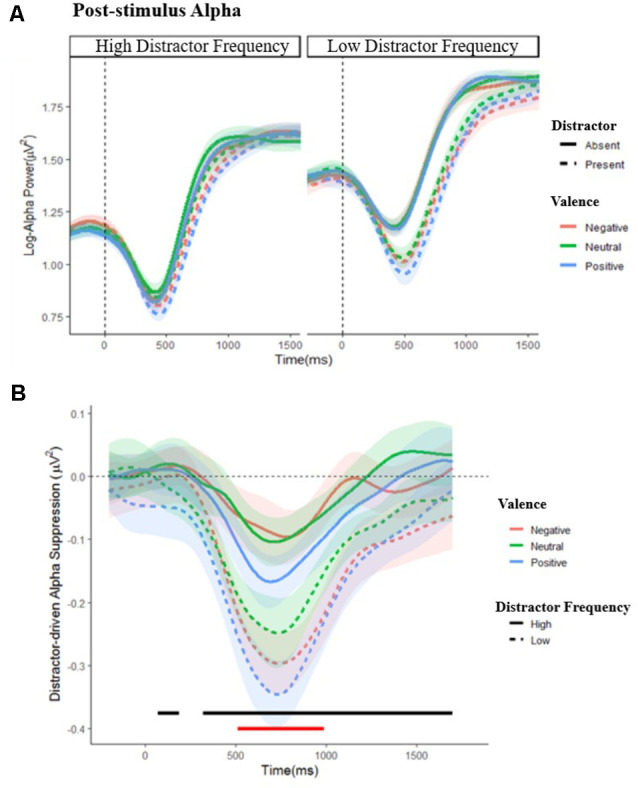
Post-stimulus alpha power. **(A)** Grand average waveforms of post-stimulus alpha power in the low and high distractor frequency conditions, following either a distractor (dashed lines) or no-distractor (solid lines), appearing at 0 ms, by valence block. Stimulus onset occurs at 0 ms. **(B)** Distractor driven alpha suppression (calculated as Distractor Present − Distractor Absent alpha power) in the low (dashed lines) and high (solid lines) distractor frequency conditions. The black line indicates where distractor-driven alpha suppression was greater in the low compared to the high distractor frequency condition. The red line indicates where there was a main effect of valence: alpha was more suppressed following emotional compared to non-emotional distractors during this period. Shaded error bars depict the ±95% within-subjects confidence intervals.

Dynamic changes in post-stimulus alpha are shown in Figure 5; a complete set of figures showing the *F* and FDR-corrected *p* values for all effects across the time window appears in Supplementary Figure S2. A main effect of distractor presence showed, as expected, greater suppression following stimulus onset when distractors were present vs. absent; this effect was significant from 288 to 1,588 ms post-stimulus, *F*’s = 4.102–188.134, *p*’s = 0.001–0.050. Importantly, this effect interacted with distractor frequency, *F*’s = 4.01–9.12, *p*’s = < 0.001–0.050, showing that distractor-driven alpha suppression (i.e., the greater suppression that occurs following the appearance of distractors) was more pronounced in the low compared to high distractor frequency condition. Further analysis of this distractor-driven alpha suppression revealed a main effect of valence from 512 to 990 ms, *F*’s = 4.64–6.39, *p*’s = 0.021–0.050, which did not interact with distractor frequency, *F*’s = 0.169–2.280, *p*’s ≤ 0.983. To clarify the effect of valence, a further ANOVA was conducted on the mean suppression indices within this window. A significant quadratic effect of valence, *F*_(1,57)_ = 10.154, *p* = 0.002, $\eta_{\text{p}}^{2}$ = 0.151, showed greater distractor-driven alpha suppression following emotional than neutral distractors.

## Discussion

Replicating previous findings (Grimshaw et al., 2018; Micucci et al., 2020), emotional images were more distracting than neutral ones when distractors were rare, but not when they were frequent. A parsimonious explanation of these findings is that greater proactive control is engaged when distractors are expected to appear more frequently, which enables effective control of emotional distractors. Our aim here was to test this account directly by measuring alpha suppression as an online index of cognitive control. In discussing these findings, we return to our four questions. First, does high distractor frequency promote the use of either sustained or dynamic proactive control? Second, is such control tailored to the expected valence of a distractor? Third, is proactive control achieved through location-based distractor inhibition? Finally, how does high distractor frequency affect the subsequent neural response to distractors?

### Does High Distractor Frequency Promote the Use of Proactive Control?

In answer to the first question, alpha was tonically suppressed in the high compared to low distractor frequency condition, indicating greater baseline attentional engagement when distractors were expected to appear often, consistent with the use of sustained (i.e., block-wide) proactive control. Because proactive control requires continuous goal maintenance, the sustained increase in attentional engagement under high distractor frequency is consistent with predictions from the DMC (Braver, 2012). Indeed, in other paradigms, neural indices of attentional engagement have shown similarly sustained differences between proactive and reactive control conditions (Chiew and Braver, 2013; Marini et al., 2016), and proactive control can be mediated by sustained activation in the lPFC (Jimura et al., 2010; Lesh et al., 2013). A sustained proactive strategy in the high distractor frequency condition is also consistent with findings from the list-wise manipulations of trial proportion in other paradigms (e.g., Stroop, flanker), which have been found to promote proactive control that is sustained across trials in anticipation of upcoming conflict (Bugg and Crump, 2012).

We also observed an expected phasic suppression of alpha following fixation onset, but the degree of suppression did not differ according to either valence or distractor frequency. The similar pattern of phasic alpha suppression across conditions suggests that, on a trial-by-trial basis, participants prepared similarly to attend to the target regardless of their expectations about upcoming distractors. This lack of difference in phasic alpha suppression between conditions further supports the conclusion that the effective control of distraction in the high distractor frequency condition is achieved through a sustained, rather than dynamic, proactive strategy.

The DMC framework suggests that sustained proactive control is implemented through the alteration of attentional control settings to optimize task-relevant processing, and our data are consistent with this hypothesis. However, we must acknowledge an alternative mechanism, that the apparent block-wide alpha suppression in the high compared to low distractor frequency condition instead reflects an accumulation of sequential effects. Alpha suppression following presentation of a distractor may carry over into the next trial, and this carryover would occur more frequently in the high distractor frequency condition. Note that this is an alternative mechanism of proactive control, reflecting a transient up-regulation of control following conflict (i.e., conflict adaptation; Botvinick et al., 2001). Unfortunately, the current study was not designed to distinguish between global anticipatory control and such cumulative sequential effects, a widespread limitation in paradigms manipulating conflict proportions. In order to clearly disentangle global anticipatory from sequential effects, future studies will need to ensure sufficient trials in each condition to reliably compare trials that follow distractor-present vs. distractor-absent trials. Additional EEG indices of control may also be useful in further exploring potential mechanisms. Although we confined our analysis here to posterior alpha as a well-established index of control, other measures (e.g., frontal alpha, midfrontal theta) may provide additional insights into the broader network that implements control. Regardless of the specific mechanisms by which posterior alpha suppression facilitates performance, our findings provide clear evidence that increased distractor frequency promotes proactive control.

### Is Proactive Control Tailored to the Potential Potency of an Expected Distractor?

Because proactive control is often associated with some sort of cognitive “effort,” we wondered whether participants would engage greater control (or at least greater pre-stimulus alpha suppression) when they expected distractors to be emotional. Given that emotional distractors are more disruptive in the low-frequency condition, we might expect greater proactive alpha suppression to be required to achieve their effective control in the high-frequency condition. If so, we should have seen greater pre-stimulus alpha suppression in emotional than in neutral blocks and particularly in the high distractor frequency condition. However, expected emotional (compared to neutral) distractors did not increase either tonic or phasic alpha suppression. This means that the same level of alpha suppression was sufficient to guard against both emotional and neutral distractors in the high-frequency condition. We speculate that the control of emotional distraction may depend on a threshold level of alpha suppression. Alpha power above threshold may allow distractors to capture attention, requiring reactive control mechanisms that are less effective against emotional than neutral distractors. However, alpha power sustained below threshold may effectively guard against all distractors regardless of valence. In other words, the emotionality of a distractor becomes relevant *after* it has captured attention, but not before.

### Is Proactive Control Implemented *via* Suppression of Potential Distractor Locations?

Proactive control describes a collection of possible mechanisms that can act prior to conflict to reduce its impact. One possible mechanism by which proactive control might be implemented in our task is through anticipatory inhibition of processing in areas of the visual field where distractors frequently appear (Wang and Theeuwes, 2018a,b). We therefore blocked the visual field in which a distractor could appear so that we could examine lateralized alpha suppression, a measure that has been proposed as an index of spatial inhibition of visual processing (Foxe and Snyder, 2011). We found no systematic alpha lateralization in any condition, and therefore no evidence that anticipatory spatial inhibition served as a mechanism of proactive control. On the face of it, this finding suggests that proactive control might be implemented through the enhancement of visual processing related to the target (either its location or features), and not suppression related to distractors. However, recent evidence cautions that location-based distractor inhibition can occur in the absence of alpha lateralization (e.g., Noonan et al., 2016), and the validity of alpha lateralization as an index of spatial inhibition has recently been challenged (Foster and Awh, 2019). Therefore, our failure to find alpha lateralization should not be taken as strong evidence of whether participants did (or did not) use location-based inhibition as a mechanism of control. Future research that can dissociate target enhancement from distractor suppression (for example, using the N2pc and Pd components in ERP studies; Hickey et al., 2009; Hilimire et al., 2012) will be important in elucidating the specific mechanisms by which proactive control is implemented.

### How Does High Distractor Frequency Affect the Neural Response to Distractors?

In both distractor frequency conditions, post-stimulus alpha suppression was greater following distractors (relative to when targets were presented alone) and was more pronounced when the distractors were emotional. This pattern of findings is in line with previous reports that alpha suppression is greater following high compared to low conflict stimuli (Itthipuripat et al., 2017; Jiang et al., 2018a). Importantly, there was less distractor-driven suppression in the high compared to the low distractor frequency condition, suggesting that distractors produced less conflict when they were expected to appear frequently. Although proactive and reactive control are commonly taken to be independent (Braver, 2012; Mäki-Marttunen et al., 2019) and implemented through different neural systems (Braver et al., 2007; Geng, 2014; Schmid et al., 2015), they may still exhibit a reciprocal relationship, in that greater proactive control reduces the need for subsequent reactive control.

## Conclusion

Emotional stimuli are, of course, everywhere. Although they often provide important survival information, they can also be entirely irrelevant to our current goals. Our findings here replicate previous research showing that our sensitivity to emotional distractors depends on our current mode of cognitive control: when conditions favor reactive control, task-irrelevant emotional images disrupt performance, but when conditions favor proactive control, neither emotional nor non-emotional images are distracting. Such flexible use of cognitive control allows for an optimal response to emotional distractors. When they are rare, they may signal important survival information and so it is adaptive for them to interrupt ongoing processing. However, when they appear frequently without consequence, they are no longer informative, and effective goal-directed behavior is best served if they are ignored. Here, we provide evidence that top-down modulation of posterior alpha power may be one mechanism by which these dual modes of emotional control can be implemented.

## Data Availability Statement

Deidentified data, analysis scripts, and experiment materials are available at: https://osf.io/7ztkw/?view_only=a2c72b64d7dd4708b8f2f47d8626ee59.

## Ethics Statement

The studies involving human participants were reviewed and approved by the Human Ethics Committee of the School of Psychology, Victoria University of Wellington. The patients/participants provided their written informed consent to participate in this study.

## Author Contributions

JM and GG conceived the research question. All authors contributed to the research design. JM and CD programmed the experiment. JM collected and analyzed data and wrote the first draft of the manuscript. All authors contributed to the article and approved the submitted version.

## Conflict of Interest

The authors declare that the research was conducted in the absence of any commercial or financial relationships that could be construed as a potential conflict of interest.

## Footnotes

^1^The neutral condition was entered as the middle level of the valence variable, meaning that differences between emotional and neutral conditions (regardless of valence) would be revealed as quadratic effects.

^2^Because overall response times were slower (non-significantly) in the high-frequency condition (see Table 1), we repeated this analysis using a proportional measure of distraction [(RT distractor present − RT distractor absent)/(RT distractor absent)]. This normalization did not change any of the reported effects (see Supplementary Material 1.4).
