# Supplementary material for: Proactive Control of Emotional Distraction: Evidence From EEG Alpha Suppression
Source: Front Hum Neurosci. 2020 Aug 18;14:318. doi: 10.3389/fnhum.2020.00318 (PMC7461792; doi:10.3389/fnhum.2020.00318)
Supplement: Supplementary file 1 [file Table_1.docx]

Supplementary Material

# Supplementary Data

## IAPs image set

**For women:**

Neutral – 2026; 2102; 2221; 2305; 2393; 2397; 2411; 2512; 2593; 2595; 2745.1; 2840;

Negative – 3015; 3030; 3059; 3103; 3131; 3140; 3150; 3195; 3550.1; 9253; 9405; 9420;

Positive – 4658; 4659; 4660; 4668; 4680; 4690; 4693; 4694; 4695; 4697; 4698; 4800.

**For men:**

Neutral – 2026; 2102; 2104; 2221; 2393; 2397; 2411; 2512; 2593; 2595; 2745,1; 2840;

Negative - 3000; 3015; 3053; 3060; 3069; 3071; 3080; 3100; 3120; 3130; 3131; 9410;

Positive – 4645; 4650; 4653; 4658; 4660; 4666; 4669; 4680; 4690; 4692; 4693; 4698.

**1.2 Resting Alpha Group comparison**

To compare group levels of alpha prior to beginning the experiment, we conducted an FFT on the resting EEG activity across the 2-minute pre-experimental resting period, and compared mean activity in the 8-12Hz band at electrode Oz using an independent samples t-test. There was no difference in alpha power between the low (*M* = 10.6, *SE* = 2.23) and high (*M* = 10.8, *SE* = 2.08) frequency conditions prior to the experiment, *t*(57) = .086, *p* = .932, indicating that the tonic differences in alpha observed during the task are accounted for by our frequency manipulation, and not pre-existing differences.

**1.3 Behavioural RT Analyses**

Mean RTs were entered in a 3 (valence) x 2 (distractor presence) x 2 (distractor frequency: low, high) mixed ANOVA. There was no main effect of distractor frequency, F(1, 57) = .056, p = .813, ηp2 = <.01, showing there was no overall difference in reaction time between conditions.

A main effect of distractor presence, *F*(1, 57) = 13.42, *p* = < .001, *η_p_^2^* = .19, revealed responses were slower when distractors appeared. There was no main effect of valence *F*(2, 114) = 0.663, *p* = .493, *η_p_^2^* = .011, and no valence x distractor presence interaction, *F*(1, 57) = 0.66, *p* =.295, *η_p_^2^*= .01, but there was a distractor presence x distractor frequency interaction, *F*(1, 57) = 15.65, *p* = < .001, *η_p_^2^* = .22, showing responses were slowed more by distractors in the low than in the high distractor frequency condition. All effects were qualified by the predicted, but not significant, three-way valence x distractor presence x distractor frequency interaction, *F*(1.71, 100.11) = 2.78, *p* = .053, *η_p_^2^* = .05. Although not significant, this three-way interaction has now been replicated in this paradigm several times (Grimshaw et al, 2015; Kranz, 2015), and therefore likely reflects greater effect of distractor frequency on the impact of emotional than of neutral distractors.

**1.4 Normalised Distraction Indices**

Distraction Indices were calculated using the formula [(RT distractor present - RT distractor absent)/(RT distractor absent).The 2-way mixed ANOVA of distractor frequency x valence (see Figure 2) showed the predicted main effect of distractor frequency, F( 1, 57) = 15.00, p < .001, *η_p_^2^*= .208; distraction was attenuated in the high compared to low distractor frequency condition, consistent with the predictions of the DMC framework. There was no main effect of valence, *F*(1.743, 99.34) = 0.681, *p* = .508, *η_p_^2^*= .012. However, the predicted interaction between distractor frequency and the quadratic effect of valence was marginally significant, *F*(1, 57) = 3.979, *p* = .051, *η_p_^2^* = .065. Replicating previous findings (Grimshaw et al., 2018), follow-up one way ANOVAs revealed a significant quadratic effect of valence in the low distractor frequency condition, *F*(1, 28) = 5.27, *p* = .029, *η_p_^2^* = .158, with both positive and negative images producing more distraction than neutral ones. However, there was no effect of valence nor quadratic effect of valence in the high distractor frequency condition, *F*(2, 58) = .151, *p* = .860, *η_p_^2^* = .005 , and *F*(1, 29) = .273, *p* = .605, *ηp^2^* = .009, respectively.
