# Supplementary figures and images for: Proactive Control of Emotional Distraction: Evidence From EEG Alpha Suppression
Source: Front Hum Neurosci. 2020 Aug 18;14:318. doi: 10.3389/fnhum.2020.00318 (PMC7461792; doi:10.3389/fnhum.2020.00318)

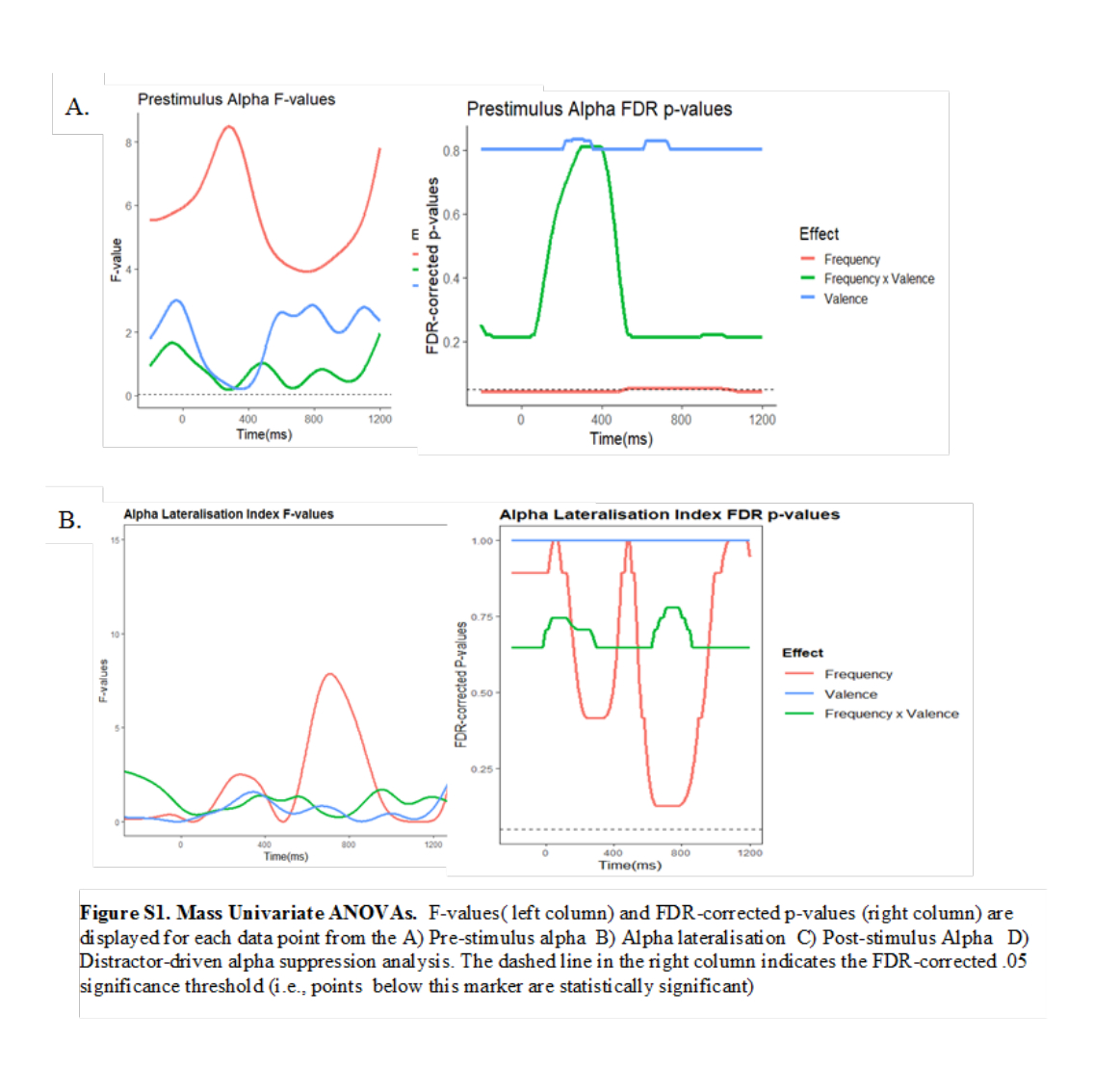

Supplement: Supplementary file 2 [file Image_1.JPEG]

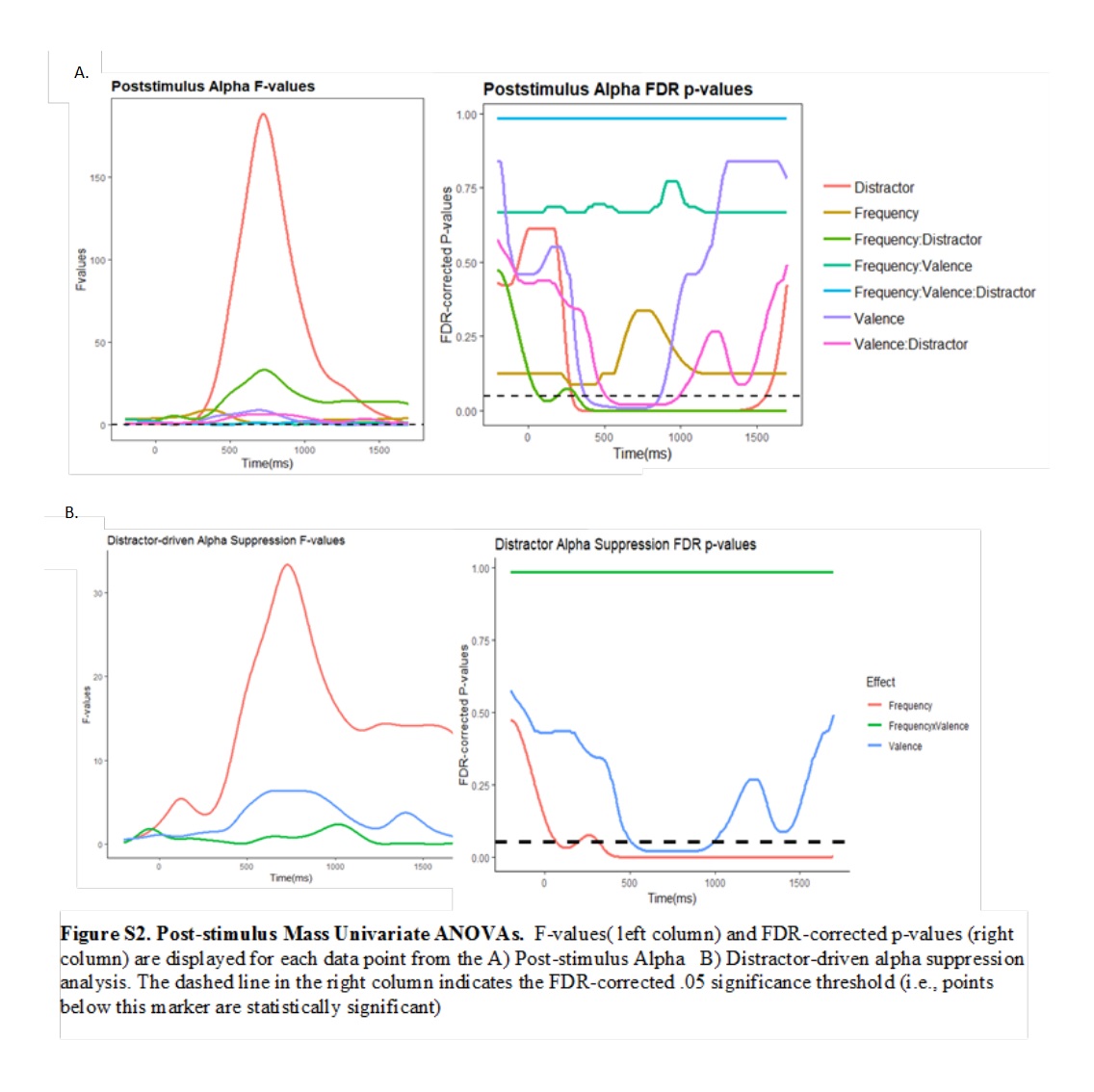

Supplement: Supplementary file 3 [file Image_2.jpg]
